# Tailored, psychological intervention for anxiety and/or depression in people with chronic obstructive pulmonary disease (COPD), TANDEM (Tailored intervention for ANxiety and DEpression Management in COPD): statistical analysis plan for a randomised controlled trial

**DOI:** 10.1186/s13063-020-04786-1

**Published:** 2020-10-15

**Authors:** Claire L. Chan, Melanie Smuk, Ratna Sohanpal, Hilary Pinnock, Stephanie J. C. Taylor, On behalf of the TANDEM Investigators

**Affiliations:** 1 Institute of Population Health Sciences, Barts and The London School of Medicine and Dentistry, Queen Mary University of London, Yvonne Carter Building, 58 Turner Street, London, E1 2AB UK; 2 London School of Hygiene and Tropical Medicine, Keppel Street, London, WC1E 7HT UK; 3 Allergy and Respiratory Research Group, Usher Institute, The University of Edinburgh, Doorway 3, Medical School, Teviot Place, Edinburgh, EH8 9AG UK

**Keywords:** Statistical analysis plan, Pragmatic randomised controlled trial, Multi-centre, Partial clustering, Internal pilot, Chronic obstructive pulmonary disease, Depression, Anxiety, Cognitive behavioural approach, Pulmonary rehabilitation, Complex intervention, Clinical effectiveness

## Abstract

**Background:**

The aim of the TANDEM trial is to evaluate whether a tailored, psychological cognitive behavioural approach intervention, which links into, and optimises the effects of routine pulmonary rehabilitation (PR), leads to a reduction in mild/moderate anxiety and/or depression in people with moderate, severe or very severe chronic obstructive pulmonary disease.

**Methods and design:**

TANDEM is a multi-centre, two-arm, parallel group, pragmatic, individually randomised controlled, superiority trial including an internal pilot. Participants are randomised to receive either the intervention (a tailored psychological intervention plus usual care including referral to PR) or the control (usual care including referral to PR). The designed randomisation ratio is 1.25:1 in favour of the intervention. The multiple-primary outcomes are participant depression and anxiety at 6 months, measured using the Hospital Anxiety and Depression Scale (HADS) depression and anxiety subscales.

**Results:**

This article describes the statistical analysis plan (SAP) for the TANDEM trial. In particular, we describe the general analysis principles, how we will handle missing data, the primary and secondary outcomes and how these will be analysed, sensitivity analyses for the multiple-primary outcomes, and any other analyses and data summaries. The SAP was developed and published prior to completion of follow-up of the last participant.

**Trial registration:**

ISRCTN registry ISRCTN59537391. Registered on 20 March 2017.

## Background

TANDEM (Tailored intervention for ANxiety and DEpression Management in chronic obstructive pulmonary disease (COPD)) is an ongoing multi-centre (twelve NHS trusts), two-arm, parallel group, pragmatic, individually randomised controlled, superiority trial including an internal pilot (*n* = 45). The aim of the pilot was to inform the feasibility of delivering the intervention, the trial processes, and progression to the main trial. No assessment of efficacy was made. The pilot was completed in 2018, and data from this will be incorporated as an internal pilot as no significant changes were made to the intervention or study procedures. The protocol for the TANDEM trial has been published previously [1] and gives details on the trial rationale, intervention and control groups, recruitment and study procedures including eligibility criteria, and the sample size calculation.

In brief, people living with COPD are at an increased risk of depression and anxiety. Pulmonary rehabilitation (PR) can help but patients commonly fail to attend or complete PR. The TANDEM trial aims to evaluate whether a tailored, psychological cognitive behavioural approach (CBA) intervention, which precedes and optimises the benefits of currently offered PR, leads to a reduction in mild/moderate anxiety and/or depression in people with moderate, severe or very severe COPD.

The planned sample size was 430 participants to achieve an expected 90% power with an estimated 20% drop out rate. Participants are randomised with a designed allocation ratio of 1.25:1 (intervention: control), using minimisation within each of the twelve NHS Trusts. Participants are allocated with probability > 1/2 to the treatment group which minimises the overall imbalance between arms at baseline for anxiety, depression, breathlessness, and smoking status based on patients already in the trial in each NHS Trust.

The intervention consists of a tailored psychological intervention based on CBA plus usual care including referral to PR, and the control is usual care (including referral to PR). ‘TANDEM Facilitators’ deliver the intervention on a one to one basis weekly for 6 to 8 weeks, depending upon the severity of the anxiety/depression and the participant’s progress. This is followed by telephone support, if the participant decides to attend PR, prior to the start of PR, during PR, and 2 weeks after completion of PR. The CBA intervention precedes PR and targets individuals’ cognitions and behaviours associated with anxiety and depression. The study has a partially clustered design in that participants are clustered by facilitator in the intervention arm only. In the control group, usual care follows arrangements for provision of the standard multidisciplinary PR programme provided in that local area. In addition, all participants also receive a publicly available British Lung Foundation (BLF) DVD: “Living with COPD”/ “Stay Well Stay Active” and a publicly available BLF COPD information and exercise and pulmonary rehabilitation booklets. Where applicable, and with participants’ permission, participants’ main carers are invited to join a sub-study to determine the effect of the intervention on carers.

The objectives of the trial are to:

1. Examine the clinical effectiveness of the CBA intervention on clinical outcomes compared to usual care.
2. Examine the process outcomes.
3. Examine the effect of the CBA intervention on carers (where appropriate).
4. Determine the cost-effectiveness of the CBA intervention from an NHS and personal social services perspective.
5. Conduct a process evaluation to inform the implementation of the CBA intervention if the trial is positive, or assist interpretation of findings if it is negative.

This paper reports on the statistical analysis plan (SAP) for the trial. The remit of the SAP covers the quantitative aspect of objectives 1, 2, and 3. Health Economic outcomes and process outcomes are addressed elsewhere.

A detailed SAP was prepared by the Pragmatic Clinical Trials Unit (PCTU) trial statistician (CC), and version 1.0 was signed off by the senior statistician (MS) on 10 February 2020 and co-chief investigators (ST and HP) on 10 and 11 February 2020. The SAP is based on protocol version 8.0 (29 May 2019). There have been no revisions to the SAP following version 1.0. All inputting members were blind to participants’ treatment group allocations.

Reporting follows the Guidelines for the Content of Statistical Analysis Plans in Clinical Trials [2] (see Additional file 1 populated checklist).

## Outcomes

### Multiple-primary outcomes

The multiple-primary outcomes are participant depression and anxiety at 6 months, measured using the Hospital Anxiety and Depression Scale (HADS) anxiety and depression subscale scores at 6 months post-randomisation [3]. There are 14 items on the HADS questionnaire, with each item scored from 0 to 3. The HADS questionnaire has 7 items related to anxiety (HADS-A) and 7 related to depression (HADS-D). Scores are totalled across the anxiety subscale to give a score for anxiety and are totalled across the depression subscale to give a score for depression. Participants can score between 0 and 21 on each subscale, with higher scores indicating worse symptoms of anxiety or depression.

### Secondary outcomes

The secondary outcomes measured in study participants include:

- Depression, measured using HADS-D, at 12 months
- Anxiety, measured using HADS-A, at 12 months
- Depression, measured using Beck Depression Inventory II (BDI-II), at 6 and 12 months [4]
- Anxiety, measured using Beck Anxiety Inventory (BAI), at 6 and 12 months [5]
- Smoking status at 6 and 12 months
- Respiratory Health-related quality of life, measured using St George’s Respiratory Questionnaire (SGRQ), at 6 and 12 months [6]
- Cognitive and emotional illness perceptions, measured using the Brief Illness Perception Questionnaire (B-IPQ), at 6 and 12 months [7]
- Social engagement, measured using the University of Melbourne Health Education Impact Questionnaire social engagement scale (heiQ), at 6 and 12 months [8]
- Social functioning, measured using an adapted version of the United Kingdom Time Use Survey, at 6 and 12 months [9]

Secondary outcomes recorded from participants’ main carers include:

- Carer burden interview, measured using the Zarit Caregiver Burden Inventory (ZBI), at 6 and 12 months [10]
- Carer mental well-being, measured using the Warwick Edinburgh Mental Well-Being Scale (WEMWBS), at 6 and 12 months [11]

Reliability indicators for instruments can be found within the references given for each instrument. See Table 1 for information on timing of data collection and Additional file 2 for information on how outcomes are derived. Health Economic outcomes (Participant Quality of life (EQ-5D-5L), Client Service Receipt Inventory, and Health Care Resource Use), and process outcomes, are addressed elsewhere.

**Table 1:** Study data collection

Type of data	Time of data collection^a^	Source and method of data collection	Outcome measure and type
**Patient participants**			
Demographics	Baseline	Patient participants; supervised self-complete questionnaire	
Clinical: Breathlessness	Baseline	Patient participants; supervised self-complete questionnaire	mMRC Breathlessness scale; categorical
Clinical: Smoking status	Baseline 6 months 12 months	Patient participants; supervised self-complete questionnaire	Smoking status; categorical
Health status measures	Screening 6 months 12 months	Patient participants; supervised self-complete questionnaire	HADS-A and HADS-D; continuous
	Baseline 6 months 12 months	Patient participants; supervised self-complete questionnaire	BDI II, BAI, B-IPQ, SGRQ, heiQ, Time Use Survey (adapted); continuous
PR attendance and completion data	Once following completion of intervention delivery	PR service teams	Attendance and completion data
CBA attendance and completion data	Attendance or failure to deliver recorded at each session during CBA intervention period	Study team/CBA facilitators	Attendance and completion data
**Carer participants**			
Demographics	Baseline	Carer; self-complete questionnaire	
Wellbeing measures	Baseline 6 months 12 months	Carer; self-complete questionnaires	ZBI, WEMWBS; continuous

^a^Note that for participant 6- and 12-month assessment, every effort is made to collect data at the scheduled time period, but in some cases, follow-up period may need to be extended ± 4 weeks for logistical/practical reasons

*Abbreviations*: *HADS* Hospital Anxiety and Depression Scale (*D* depression sub-scale, *A* anxiety subscale) [3], *mMRC* modified MRC breathlessness scale [12], *BAI* Beck Anxiety Inventory [5], *BDI II* Beck Depression Inventory II [4], *SGRQ* St George’s Respiratory Questionnaire [6], *heiQ* University of Melbourne Health Education Impact Questionnaire social engagement scale [8], *B-IPQ* The brief illness perception questionnaire [7], *ZBI* Zarit Caregiver Burden Inventory [10], *WEMWBS* Warwick Edinburgh Mental Well-Being Scale [11]

## Analysis methods

### General analysis principles

Analyses will follow the intention-to-treat principle. This requires that all participants be included in the analysis according to the treatment group to which they were randomised, regardless of any departures from randomised treatment [13]. Where it is not possible to follow up participants, we will handle missing data by including all those with a recorded outcome [14].

Analyses will be presented as:

- The number of participants included in the analysis, by treatment group
- A summary measure of the outcome, by treatment group (e.g. mean (standard deviation) for continuous outcomes)
- A treatment effect (e.g. difference in means for continuous outcomes) with a 95% confidence interval
- A two-sided *p* value

For all analyses, a significance level of 5% will be used, except when the Hochberg procedure is applied to account for there being two primary outcomes. We will use a Hochberg procedure to analyse the two primary outcomes [15]. Briefly, the Hochberg procedure states that if either outcome has a *p* value < 0.025 then that outcome is statistically significant; additionally, both outcomes are significant if the *p* values are both < 0.05. We assume that the positive dependence assumption of the Hochberg correction holds. *p* values will be computed using the Satterthwaite approximation method [16].

All analyses will account for the partially clustered design of the study, where participants are clustered by facilitator in the intervention arm only. Each participant in the intervention arm will be defined as belonging to a cluster, defined by which facilitator they belonged to. It is possible that the facilitator could change for a participant, for example if a facilitator withdraws and a new facilitator is assigned to a participant. In such scenarios, for analysis purposes, we will consider that the participant is clustered within the facilitator for which they have had the majority of their sessions. If they had equal amounts of sessions under multiple facilitators, we will take the first of such facilitators. The clustering effect by facilitator in the intervention arm will be modelled using a partially nested mixed-effects model, which confines the random effect for cluster to the intervention arm only. This is achieved by fitting a random slope for treatment group while suppressing the constant from the random part. This essentially amounts to a random intercept for each cluster in the intervention arm and one intercept for the unclustered control arm [17, 18]. In order to run the model in Stata, it is necessary to impose clustering in the control arm. We therefore will treat each participant in the control arm as a single cluster to facilitate modelling [18].

All analyses will also account for the correlation between outcomes at 6 and 12 months using a random effect for participant. This approach will provide unbiased estimates even if some participants only provide data at one of the two time points, under the missing at random assumption implied by the model. We fit a heteroscedastic model, allowing for different residual level errors in the two treatment arms since we expect participants in the intervention arm to vary in a different way to those in the control arm [17]. The heteroscedastic partially nested mixed-effects model is recommended in this setting [18], and by implementing the method, we could possibly yield insight about the treatment [19]. The model will be estimated using restricted maximum likelihood (REML). Treatment arm, time point (month 6 or 12), and the interaction between treatment arm and time point will be included in the model as fixed factors.

All analyses will adjust for the outcome measured at baseline whenever possible. Moreover, analyses will reflect the design of the study, and so the stratification and minimisation variables will be adjusted for [20]. These covariates will not be interacted with time point. Continuous covariates will be fitted for the HADS-A and HADS-D screening scores. Although categories were used for HADS-A and HADS-D during the minimisation, we avoid unnecessary categorisation of the covariates for the analysis as this should increase power [21]. Continuous covariates will be assumed to have a linear relationship with the outcome. Binary covariates will be fitted for the baseline degree of breathlessness (categories, 0–2 and 3–4) and baseline smoking status (categories, smoker and non-smoker (ex-smoker/never smoked)). We will also adjust for NHS Trust as a fixed categorical variable instead of a complex (four-level) mixed effect model due to concerns about model fitting within the sample size. Furthermore, when there are a large number of participants per Trust (25 or more, which we expect here), random and fixed effects perform equally well in terms of coverage, power, and efficiency [22, 23].

All outcomes are continuous except for the binary outcome smoking status at 6 and 12 months (current smoker vs non-smoker). Since some participants will already be non-smokers at baseline, for this outcome, we will present a simple comparison of proportions between baseline, 6 months, and 12 months. Other analyses involving binary outcomes, such as CBA intervention attendance and completion rates, and PR attendance and completion rates, are discussed later.

Analyses will be carried out using Stata version 14 or newer. R version 3.6.1 or newer may be used if necessary. The software and version number used will be referenced with any analysis write up.

See Additional file 3 for table shells and a CONSORT flow diagram shell.

### Missing data

We do not expect missing data for any of the baseline covariates for the primary analysis. It is possible that there will be a small amount of missing data for the secondary outcomes measured at baseline. In this case, missing data for baseline covariates to be included in the analysis model will be accounted for using mean imputation [24].

For outcomes that are measured at multiple time points during follow-up, we have based our analysis strategy on that proposed by White et al. [14]. To deal with incomplete data (i.e. when participants have missing data at one of the follow-up time points), we will:

1. Attempt to follow up all randomised participants even if they withdraw from the allocated treatment (but remain in the study).
2. Perform a main analysis of all observed data that are valid under a plausible assumption about the missing data.
3. Perform sensitivity analyses to explore the effect of departures from the assumptions made in the main analysis.
4. Account for all randomised participants, at least in the sensitivity analyses.

For the main analyses (point 2), we will include all participants with at least one post-randomisation assessment (i.e. if the relevant outcome is recorded for at least one follow-up time point) in the analysis. Participants with missing outcome data at both 6 and 12 months will be excluded for this analysis. The mixed-effects model adjusted for baseline covariates assumes that the data are missing at random (MAR) [25]. Modelling of the observed data in this way is a principled method to deal with missingness, as information is ‘borrowed’ from other clusters. For outcomes that consist of several items combined to create a score, we expect most participants will either complete all or none of the items as observed in the trial data monitoring. We will thus consider the summary outcome score as missing if any item is missing. Only participants who completed all of the questions which form the score at either 6 or 12 months will be included in the analysis.

We will perform sensitivity analyses for the primary outcomes to assess the robustness of our primary analysis to the missing data assumptions (point 3) and account for all randomised participants including those lost to follow up, withdrawn, or found to be ineligible after randomisation (point 4). We will also compare the distribution of baseline characteristics of people included in the primary analysis model and those missing the primary outcome at both 6 and 12 months. Moreover, we will present the characteristics of people who have missing follow-up data because they died.

### Analysis of multiple-primary outcomes

We will analyse the multiple-primary outcomes separately. This is equivalent to fitting a joint model in the case of no missing data. A joint model would be more efficient in the case where one outcome is missing but the other outcome is not. However, a joint model is more computationally complex and it is unlikely that participants would have completed the HADS-A questions but not the HADS-D, and vice versa.

A heteroscedastic partially nested mixed-effects model will be fitted for HADS-A and HADS-D, as follows:

$$ \mathrm{Outcome}={\upbeta}_0+{\upbeta}_1\mathrm{treat}+{\upbeta}_2\mathrm{time}+{\upbeta}_3\mathrm{treat}\ast \mathrm{time}+{\upbeta}_4\mathrm{trust}2+\dots +{\upbeta}_{14}\mathrm{trust}12+{\upbeta}_{15}\mathrm{hadsa}+{\upbeta}_{16}\mathrm{hadsd}+{\upbeta}_{17}\mathrm{breath}+{\upbeta}_{18}\mathrm{smoke}+{\mathrm{u}}_2\mathrm{treat}+{\mathrm{u}}_1+\upvarepsilon $$

where:

treat = Treatment allocation = 0 if control; 1 if intervention

time = Measurement occasion = 0 if 6 months; 1 if 12 months

trust = NHS Trust = 1 if trust 1; 2 if trust 2; …; 12 if trust12 (assuming 12 trusts)

hadsa = Baseline HADS-A = (continuous)

hadsd = Baseline HADS-D = (continuous)

breath = Baseline breathlessness = 0 if “0–2”; 1 if “3–4”

smoke = Baseline smoking status = 0 if non-smoker; 1 if smoker

u_2_ = Facilitator random effect

u_1_ = Participant random effect

ε = Residual error

u_2_|covariates ~ *N* (0, σ^2^_u2_)

u_1_|covariates, u_2_ ~ *N* (0, σ^2^_u1_)

ε|(treat = 0) ~ *N* (0, σ^2^_ε0_)

ε|(treat = 1) ~ *N* (0, σ^2^_ε1_)

### Sensitivity analyses for multiple-primary outcomes

#### Missing data

For the primary analysis, we do not expect missing data for any of the baseline covariates because they are all randomisation variables. However, there may be some missing outcome data which we assume is missing at random (MAR). MAR is assumed as the most probable mechanism as the protocol restricts the likelihood of missing data occurring from a missing not at random mechanism. Only participants who complete all of the questions that form the score at either 6 or 12 months are included in the primary analysis; participants with missing outcome data at both 6 and 12 months are excluded. This mixed-effects model adjusted for baseline covariates assumes the data are MAR.

We will perform the following sensitivity analyses for the multiple-primary outcomes to assess the robustness of our primary analysis to different assumptions regarding the missing data:

- A complete case analysis which assumes data missing at 6 months is missing completely at random (MCAR)
- An analysis which assumes that data missing at 6 months is missing not at random (MNAR)

For the complete case analysis, we will fit the primary analysis model but only include participants with fully recorded data at 6 months. Participants who did not complete all components of the HADS-A or HADS-D questions respectively at 6 months will be excluded from the corresponding analysis.

For the second analysis, we will assess the primary outcomes under a range of MNAR scenarios. This will be done following the simple approach proposed by White et al. [26]. We use the formula ∆ = ∆CC + Y1P1 − Y2P2, where ∆ is the treatment effect under the MNAR scenario, ∆CC is the treatment effect from the complete case analysis on 6-month data above, Y1 and Y2 are the assumed 6-month mean responses for participants with missing data in treatment groups 1 and 2 respectively, P1 and P2 are the proportion of participants who were excluded from the 6-month analysis in groups 1 and 2 respectively, and groups 1 and 2 represent the intervention and control groups respectively. The standard error for ∆ is assumed to be approximately equal to the standard error for ∆CC. Y2 will be varied for both outcomes between − 10, − 5, − 1.5, 0, 1.5, 5, and 10. Negative values indicate the participant got less anxious/depressed at 6 months, positive values indicate they got more so, and a value of 0 indicates there was no change from baseline. For each value of Y2, Y1 will be set to Y2 − 5, Y2, and Y2 + 5. For example, for Y2 = 10 and the HADS-A outcome, this would indicate an assumption that participants in treatment arm 2 (the control arm) who were lost to follow-up at 6 months had gained 10 points on the HADS-A subscale on average at 6 months. Y1 would vary between 5, 10, and 15, indicating the assumption that participants in treatment arm 1 (the intervention arm) who were lost to follow-up had gained 5 points on the HADS-A subscale on average at 6 months (5 points less than those in the control arm), 10 points (the same amount as those in the control arm), or 15 points (5 points more than those in the control arm). It is possible that the value of ∆ that we obtain may be implausible if it goes outside the score range of 0–21. In such cases, we will truncate ∆ by the scale boundary. We will note any “tipping point”—the value the treatment effect in the non-responders would need to be to change conclusions.

#### Inclusion criteria

We will perform a sensitivity analysis to assess the impact of including participants in the analysis who have less room for improvement during follow-up. The inclusion criteria state that participants must have a score of 8 or more on either the HADS anxiety or depression subscale (but not necessarily both), so some participants may have a score of < 8 on one of these subscales and therefore have less room for improvement during follow-up. We will assess the impact of including such participants, by repeating the primary analyses for HADS-A and HADS-D but excluding participants who have a score of < 8 on the HADS-A and HADS-D subscales respectively. The anxiety and depression subscales of HADS are strongly associated, [27] meaning that it is unlikely that many participants have scores a lot lower than 8 on either subscale.

#### Time to pulmonary rehabilitation

We will assess the impact of any difference in time from baseline to attending PR in the intervention and control groups. We will summarise the time to PR in the two groups, presenting the mean (standard deviation) and the median (interquartile range). Furthermore, we will include time to PR as a covariate in the primary analysis model and assess the impact.

#### Internal pilot

We will perform a sensitivity analysis excluding the internal pilot participants from the primary analysis, to assess the potential impact of a ‘learning effect’ in the pilot. For example, it is possible that the delay between completion of training and the start of intervention delivery in the internal pilot may have resulted in the treatment looking less effective in the pilot than later participants.

#### Allocation ratio

We are aware that there has been an issue in the randomisation system for a small proportion of participants, which resulted in a differing allocation ratio for very brief periods during the trial. We will therefore do a sensitivity analysis stratifying by allocation period. We will fit the primary analysis model but separately for the participants in each of the allocation periods. The results will then be combined as in a two-stage individual participant data meta-analysis [28]. We will also look at the measured characteristics in each of these groups of participants to see if there is evidence that they are different in each time period, although TANDEM is a fairly rapidly recruiting trial and we do not expect this to be the case. We will also report the balance of minimisation factors in each NHS Trust.

### Analysis of secondary outcomes

#### HADS-D at 12 months

This outcome is included in the same model as the HADS-D primary outcome.

#### HADS-A at 12 months

This outcome is included in the same model as the HADS-A primary outcome.

#### BDI-II at 6 and 12 months

These outcomes will be analysed using the same model as the primary outcomes at 6 and 12 months, with baseline BDI-II as an additional covariate in the model.

#### BAI at 6 and 12 months

These outcomes will be analysed using the same model as the primary outcomes at 6 and 12 months, with baseline BAI as an additional covariate in the model.

#### Smoking status at 6 and 12 months

Smoking status (current smoker vs non-smoker) will be presented as a simple comparison of proportions between baseline, 6 months, and 12 months.

#### SGRQ at 6 and 12 months

Separate models will be fitted for the three component scores (symptoms, activity, impact) and the total score. These outcomes will be analysed using the same model as the primary outcomes at 6 and 12 months, with the relevant component score or total score for SGRQ at baseline as an additional covariate in the model.

#### B-IPQ at 6 and 12 months

There are 8 items, with each item scored from 0 to 10. Each item of the Brief IPQ assesses one dimension of illness perceptions. Separate models will be fitted for the 8 scores. These outcomes will be analysed using the same model as the primary outcomes at 6 and 12 months, with the relevant component score for B-IPQ at baseline as an additional covariate in the model.

#### heiQ at 6 and 12 months

These outcomes will be analysed using the same model as the primary outcomes at 6 and 12 months, with baseline heiQ as an additional covariate in the model.

#### Social functioning (adapted Time Use Survey) at 6 and 12 months

We will analyse the time (minutes) spent doing activities over the last 4 days. This outcome will be analysed using the same model as the primary outcomes at 6 and 12 months, with baseline time (minutes) spent doing activities over the last 4 days as an additional covariate in the model. Time spent doing activities over the last 4 days will be assumed to have a linear relationship with the outcome.

#### ZBI at 6 and 12 months

These outcomes will be analysed using the same model as the primary outcomes at 6 and 12 months, with baseline ZBI as an additional covariate in the model.

#### WEMWBS at 6 and 12 months

These outcomes will be analysed using the same model as the primary outcomes at 6 and 12 months, with baseline WEMWBS as an additional covariate in the model.

### Other analyses and data summaries

Baseline characteristics and questionnaires will be summarised for each treatment group by the mean and standard deviation or median and interquartile range for continuous variables, and the number and percentage for categorical variables.

We will report on CBA intervention attendance and completion rates and PR attendance and completion rates. For our analysis, a participant in the intervention arm is considered to have completed CBA (typically 6–8 sessions) if they complete 2 or more sessions (as per protocol, completion of two sessions considered minimum effective dose of the intervention). A participant in either arm of the study is considered to have completed PR if they complete 75% or more of their scheduled PR sessions. See Additional file 2 for information on how the rates are derived.

Finally, we will also report on completeness of data on questionnaires, and adverse and serious adverse events.

We do not foresee the need for an interim analysis.

## Trial status

At the time of first submission, the TANDEM trial was ongoing with recruitment having just finished on 19 March 2020 and with follow-up ongoing. Data collection is anticipated to be completed approximately 12 months following this date, and all outcomes will be analysed collectively after this.

## References to files

The Trial Master File is located on a shared drive within the PCTU and contains, amongst other things, the data management plan and standard operating procedures. The Statistical Master File is located on a separate shared drive within the PCTU.

## Supplementary information

**Additional file 1.** Populated SAP reporting guidelines checklist.

**Additional file 2.** Information on derived outcomes.

**Additional file 3.** Table shells and CONSORT flow diagram shell.

## Data Availability

Not applicable.

## Abbreviations

BAI: Becks Anxiety Inventory
BDI-II: Becks Depression Inventory
B-IPQ: Brief Illness Perception Questionnaire
BLF: British Lung Foundation
CBA: Cognitive Behavioural Approach
COPD: Chronic Obstructive Pulmonary Disease
DMEC: Data Monitoring and Ethics Committee
HADS: Hospital Anxiety and Depression Scale
HeiQ: Health Education Impact Questionnaire
MAR: Missing at random
MCAR: Missing completely at random
MNAR: Missing not at random
PCTU: Pragmatic Clinical Trials Unit
PR: Pulmonary rehabilitation
RCT: Randomised controlled trial
REML: Restricted maximum likelihood
SAP: Statistical analysis plan
SGRQ: St George’s Respiratory Questionnaire
TANDEM: Tailored intervention for ANxiety and DEpression Management in COPD
WEMWBS: Warwick-Edinburgh mental Wellbeing Scale
ZBI: Zarit Burden Interview
