# Supplementary material for: Tailored, psychological intervention for anxiety and/or depression in people with chronic obstructive pulmonary disease (COPD), TANDEM (Tailored intervention for ANxiety and DEpression Management in COPD): statistical analysis plan for a randomised controlled trial
Source: Trials. 2020 Oct 15;21:858. doi: 10.1186/s13063-020-04786-1 (PMC7559776; doi:10.1186/s13063-020-04786-1)
Supplement: Supplementary file 2 — Additional file 2. Information on derived outcomes. [file 13063_2020_4786_MOESM2_ESM.docx]

**ADDITIONAL FILE 2: INFORMATION ON DERIVED OUTCOMES**

**Co-primary outcomes**

*HADS questionnaire*

There are 14 items, with each item scored from 0-3. The HADS questionnaire has 7 items related to anxiety (HADS-A) and 7 related to depression (HADS-D). Scores are totaled across the anxiety subscale to give a total score for anxiety, and are totaled across the depression subscale to give a total score for depression. Participants can score between 0-21 on each subscale, with higher scores indicating higher anxiety or depression.

**Secondary outcomes**

*BDI-II*

There are 21 items, with each item scored from 0-3. The BDI–II is scored by summing the ratings for the 21 items. The minimum score is zero and the maximum total score is 63. Total scores from 0 to 13 reflect minimal depression; 14 to 19 reflect mild depression; 20 to 28 reflect moderate depression; and 29 to 63 reflects severe depression.

*BAI*

There are 21 items, with each item scored from 0-3. The BAI is scored by summing the ratings for the 21 symptoms. The minimum score is zero and the maximum score is 63. Total scores from 0 to 7 points are considered to reflect a minimal level of anxiety; scores of 8 to 15 indicates mild anxiety; scores of 16 to 25 reflect moderate anxiety; and scores of 26 to 63 indicate severe anxiety.

*Smoking status*

This is a single question asking the smoking status of the participant. The participant can choose one of three categories for their response: current smoker, ex-smoker, never smoked.

*SGRQ*

There are 17 items, each with varying scores. Three component scores are calculated for the SGRQ: Symptoms - this component is concerned with the effect of respiratory symptoms, their frequency and severity. It is calculated from the summed weights for the positive responses to questions 1-8. Activity - concerned with activities that cause or are limited by breathlessness. It is calculated from the summed weights for the positive responses to questions 11 and 15. Impacts - covers a range of aspects concerned with social functioning and psychological disturbances resulting from airways disease. It is calculated from the summed weights for the positive responses to questions 9-10, 12-14, and 16-17. A Total score is also calculated which summarises the impact of the disease on overall health status. It is calculated by summing all positive responses in the questionnaire. The component and total scores are expressed as a percentage of the weight for that component or the total weight, respectively. Scores are expressed as a percentage where 100 represents worst possible health status and 0 indicates best possible health status.

*Brief IPQ*

There are 8 items, with each item scored from 0-10. Each item of the Brief IPQ assesses one dimension of illness perceptions: The consequences score is simply the response to item 1. The timeline score is the response to item 2. The personal control scores is the response to item 3. The treatment control score is the response to item 4. The identity score is the response to item 5. Illness concern is measured by item 6. The coherence score is the response to item 7. The emotional representation is the response to item 8.

*heiQ*

There are 5 items on the social integration and support construct of the heiQ questionnaire, each scored from 1-4 (1 = strongly disagree, 2 = disagree, 3 = agree, 4 = strongly agree). The summary score is obtained by summing the scores for all items and dividing by the number of items. The score therefore ranges between 1 and 4, and a higher score indicates better self-management.

*Time use survey*

There are 12 items, each with a yes/no response about whether the participant has done that activity. There are further sub questions if the answer is yes, which ask the number of times the activity has been done, amount of time, whether it was done with someone, and if so who.

*ZBI*

There are 22 items, with each item scored from 0-4. Items 1 to 21 are scored as 0 = never and 4 = nearly always, and item 22 is scored as 0 = not at all and 4 = extremely. Scores are summed to give a global score, with a range of 0 to 88.

*WEMWBS*

There are 14 items, with each item scored from 1-5. A total score is calculated by summing the 14 individual statement scores. The minimum score is 14 and the maximum is 70.

**Other summaries**

*Compliance with the intervention*

We will report on CBA intervention attendance and completion rates, and PR attendance and completion rates. We will calculate the rates as follows:

CBA intervention attendance rate = a/b

CBA intervention completion rate = c/a

PR intervention attendance rate = d/e

PR intervention completion rate = f/d

Where: a = number who attended/received at least one CBA session; b = number allocated to intervention; c = number who completed CBA; d = number who attended/received at least one PR session; e = number referred and deemed suitable to attend PR; f = number who completed PR.
