# Supplementary material for: Tailored, psychological intervention for anxiety and/or depression in people with chronic obstructive pulmonary disease (COPD), TANDEM (Tailored intervention for ANxiety and DEpression Management in COPD): statistical analysis plan for a randomised controlled trial
Source: Trials. 2020 Oct 15;21:858. doi: 10.1186/s13063-020-04786-1 (PMC7559776; doi:10.1186/s13063-020-04786-1)
Supplement: Supplementary file 3 — Additional file 3. Table shells and CONSORT flow diagram shell. [file 13063_2020_4786_MOESM3_ESM.docx]

**ADDITIONAL FILE 3: TABLE SHELLS AND CONSORT FLOW DIAGRAM SHELL**

**CONSORT flow diagram**

## Screening

Number of possible participants identified by clinical team (n= )

Excluded (n= )

♦  Not contactable after identification (n= )

♦  Not wanting researcher to contact them further (n=)

Number of possible participants who agree to researcher contacting them to discuss study further (n= )

Excluded (n= )

♦  Not interested in taking part in study (n=)

♦  Not wanting to be screened (n=)

♦  Other reasons (n= )

Screened for eligibility (n= )

Allocated to PR usual care plus BLF leaflets/DVD (n= )

## Allocation

## Enrolment

Eligible (n= )

Excluded (n= )

♦  Declined to consent (n= )

♦  Other reasons (n= )

Recruited (n= )

Randomised (n= )

Allocated to CBA intervention plus PR usual care (n= )

Attended / received at least one CBA session (n= )

Completed CBA (2 or more sessions) (n= )

Referred to attend PR (n=)

Attended / received at least one PR session (n= )

Completed PR (75% or more of their scheduled PR sessions) (n= )

Referred to attend PR (n=)

Attended / received at least one PR session (n= )

Completed PR (75% or more of their scheduled PR sessions) (n= )

## Follow-Up

Lost to follow-up/withdrew/ ineligible by 6 months (n= )

Lost to follow-up/withdrew/ ineligible by 12 months (n= )

Discontinued PR (n= )

Lost to follow-up/withdrew/ineligible by 6 months (n= )

Lost to follow-up/withdrew/ineligible by 12 months (n= )

Discontinued CBA (n= )

Discontinued PR (n= )

## Analysis

Included in HADS-A primary outcome model (n= )

Number with a 6-months HADS-A (n= )

Included in HADS-D primary outcome model (n= )

Number with a 6-months HADS-D (n= )

Included in HADS-A primary outcome model (n= )

Number with a 6-months HADS-A (n= )

Included in HADS-D primary outcome model (n= )

Number with a 6-months HADS-D (n= )

**Table shells**

Table 1 - Baseline participant demographics and clinical history

|  | **Complete data** | | **Summary measure** | |
| --- | --- | --- | --- | --- |
|  | **Intervention no. (%)** | **Usual care**  **no. (%)** | **Intervention (n=…)** | **Usual care (n=…)** |
| Age (years) – mean (SD) median (IQR) |  |  |  |  |
| Male - no. (%) |  |  |  |  |
| Marital status – no. (%) |  |  |  |  |
| Lives alone |  |  |  |  |
| Lives with spouse or partner |  |  |  |  |
| Lives with adult family member |  |  |  |  |
| Lives with spouse or partner and adult family member |  |  |  |  |
| Currently in paid employment/working – no. (%) |  |  |  |  |
| (If yes) Hours per week in paid employment/working – mean (SD) median (IQR) |  |  |  |  |
| Had formal education – no. (%) |  |  |  |  |
| (If yes) Age when completed full-time education (years) – no. (%) |  |  |  |  |
| 12 years or under |  |  |  |  |
| 13-16 |  |  |  |  |
| 16-18 |  |  |  |  |
| Over 18 years |  |  |  |  |
| On home oxygen – no. (%) |  |  |  |  |
| (If yes) Hours per day on home oxygen – mean (SD) median (IQR) |  |  |  |  |
| (If yes) Type of oxygen equipment used – no. (%) |  |  |  |  |
| Concentrator |  |  |  |  |
| Large cylinder |  |  |  |  |
| Ambulatory |  |  |  |  |
| Age when first diagnosed with COPD – mean (SD) median (IQR) |  |  |  |  |
| Other long-term health problems – no. (%) |  |  |  |  |
| Heart disease |  |  |  |  |
| Diabetes |  |  |  |  |
| Arthritis |  |  |  |  |
| High blood pressure |  |  |  |  |
| Asthma |  |  |  |  |
| Epilepsy |  |  |  |  |
| Other |  |  |  |  |
| Attended a pulmonary rehabilitation course before – no. (%) |  |  |  |  |
| (If yes) Able to complete the course – no. (%) |  |  |  |  |
| Smoking status – no. (%) |  |  |  |  |
| Current smoker |  |  |  |  |
| Ex-smoker |  |  |  |  |
| Never smoked |  |  |  |  |
| (If Current Smoker) Pack years* – mean (SD) median (IQR) |  |  |  |  |
| Current vaper (inc. e-cigarette user) – no. (%) |  |  |  |  |
| Degree of breathlessness (mMRC breathlessness scale) – no. (%) |  |  |  |  |
| Not troubled by breathlessness except on strenuous exercise |  |  |  |  |
| Short of breath when hurrying on the level *or* walking up a slight hill |  |  |  |  |
| Walks slower than most people of the same age on the level because of breathlessness *or* has to stop the breath when walking at own pace on the level |  |  |  |  |
| Stops the breath after walking about 100 yards *or* after a few minutes on the level |  |  |  |  |
| Too breathless to leave the house *or* breathless when dressing or undressing |  |  |  |  |
| Participant had a recent hospitalisation (within last 6 months) for an acute exacerbation of COPD at time of pre-screening† |  |  |  |  |

**Abbreviations**: **COPD**: chronic obstructive pulmonary disease

*Pack years is calculated according to the pack years calculator at smokingpackyears.com

†This information was collected part way into the trial so will be missing for some participants

Table 2 - Baseline carer demographics

|  | **Complete data** | | **Summary measure** | |
| --- | --- | --- | --- | --- |
|  | **Intervention no. (%)** | **Usual care**  **no. (%)** | **Intervention (n=…)** | **Usual care (n=…)** |
| Age (years) – mean (SD) median (IQR) |  |  |  |  |
| Male - no. (%) |  |  |  |  |
| Relationship to participant – no. (%) |  |  |  |  |
| Son |  |  |  |  |
| Daughter |  |  |  |  |
| Other adult family member |  |  |  |  |
| Spouse or partner |  |  |  |  |

Table 3 - Baseline participant questionnaires

|  | **Complete data** | | **Summary measure** | |
| --- | --- | --- | --- | --- |
|  | **Intervention no. (%)** | **Usual care**  **no. (%)** | **Intervention (n=…)** | **Usual care (n=…)** |
| HADS (Your mood and feelings in the past week) | | | | |
| HADS-A score – mean (SD) |  |  |  |  |
| HADS-D score – mean (SD) |  |  |  |  |
| BDI-II (Your mood and feelings during the past two weeks including today) | | | | |
| Sum score – mean (SD) |  |  |  |  |
| BAI (Your worries during the past week including today) | | | | |
| Sum score – mean (SD) |  |  |  |  |
| SGRQ (Your respiratory health) | | | | |
| Total score – mean (SD) |  |  |  |  |
| Symptoms score – mean (SD) |  |  |  |  |
| Activity score – mean (SD) |  |  |  |  |
| Impact score – mean (SD) |  |  |  |  |
| B-IPQ (Your views about your illness) | | | | |
| Consequences score – mean (SD) |  |  |  |  |
| Timeline score – mean (SD) |  |  |  |  |
| Personal control score – mean (SD) |  |  |  |  |
| Treatment control score – mean (SD) |  |  |  |  |
| Identity score – mean (SD) |  |  |  |  |
| Concern score – mean (SD) |  |  |  |  |
| Coherence score – mean (SD) |  |  |  |  |
| Emotional response score – mean (SD) |  |  |  |  |
| heiQ - Social engagement (Your social life) | | | | |
| Mean score – mean (SD) |  |  |  |  |
| Time Use Survey (Activities in your spare time) | | | | |
| Time (mins) spent doing activities over last 4 days – mean (SD) median (IQR) |  |  |  |  |

**Abbreviations**

**HADS**: Hospital Anxiety and Depression Scale; **mMRC**: modified Medical Research Council; **BDI-II**: Beck’s Depression Inventory-II; **BAI**: Beck’s Anxiety Inventory; **SGRQ**: The St Georges Respiratory Questionnaire; **B-IPQ**: The Brief-Illness Perception Questionnaire; **heiQ**: The Health Education Impact Questionnaire

Table 4 - Baseline carer questionnaires

|  | **Complete data** | | **Summary measure** | |
| --- | --- | --- | --- | --- |
|  | **Intervention no. (%)** | **Usual care**  **no. (%)** | **Intervention (n=…)** | **Usual care (n=…)** |
| WEMWBS (Your feelings in the last two weeks) | | | | |
| Sum score – mean (SD) |  |  |  |  |
| ZBI (Your feelings when taking care of another person) | | | | |
| Sum score – mean (SD) |  |  |  |  |

**Abbreviations**

**WEMWBS**: Warwick-Edinburgh Mental Wellbeing Scale; **ZBI**: Zarit Burden Interview

Table 5 - Minimisation factors within each NHS Trust (separate table per trust)

|  | **Intervention** | **Usual care** |
| --- | --- | --- |
|  | **N (%)** | **N (%)** |
| **Minimisation factors** |  |  |
| **HADS-Anxiety** |  |  |
| 0 – 7 |  |  |
| 8 – 10 |  |  |
| 11 – 15 |  |  |
| **HADS-Depression** |  |  |
| 0 – 7 |  |  |
| 8 – 10 |  |  |
| 11 – 15 |  |  |
| **Modified MRC dyspnoea scale** |  |  |
| 0-2 |  |  |
| 3-4 |  |  |
| **Smoking status** |  |  |
| Smoker |  |  |
| Non-smoker (ex-smoker/never smoked) |  |  |

Table 6 - Completeness of data on questionnaires

|  | **Baseline** | | | **6 months** | | | **12 months** | | |
| --- | --- | --- | --- | --- | --- | --- | --- | --- | --- |
|  | **No. (%) giving data at all** | **No. (%) giving complete data** | **Mean (range) no. of items completed** | **No. (%) giving data at all** | **No. (%) giving complete data** | **Mean (range) no. of items completed** | **No. (%) giving data at all** | **No. (%) giving complete data** | **Mean (range) no. of items completed** |
| HADS-A (7 items) |  |  |  |  |  |  |  |  |  |
| HADS-D (7 items) |  |  |  |  |  |  |  |  |  |
| mMRC (1 item) |  |  | N/A | N/A |  |  | N/A | N/A |  |
| BDI-II (21 items) |  |  |  |  |  |  |  |  |  |
| BAI (21 items) |  |  |  |  |  |  |  |  |  |
| SGRQ (17 items) |  |  |  |  |  |  |  |  |  |
| B-IPQ (8 items) |  |  |  |  |  |  |  |  |  |
| heiQ (5 items) |  |  |  |  |  |  |  |  |  |
| Time Use Survey (12 items) |  |  |  |  |  |  |  |  |  |
| WEMWBS (14 items) |  |  |  |  |  |  |  |  |  |
| ZBI (22 items) |  |  |  |  |  |  |  |  |  |

Table 7 - Main results for analysis of primary and secondary continuous outcomes

|  | **Number included in analysis**  No. (%) | | **Summary measure**  Mean (SD) | |  | |
| --- | --- | --- | --- | --- | --- | --- |
|  | **Intervention** | **Usual Care** | **Intervention** | **Usual Care** | **Treatment effect**  **(95% CI)** | **p-value** |
| HADS-D at 6 months |  |  |  |  |  |  |
| HADS-A at 6 months |  |  |  |  |  |  |
| HADS-D at 12 months |  |  |  |  |  |  |
| HADS-A at 12 months |  |  |  |  |  |  |
| BDI-II at 6 months |  |  |  |  |  |  |
| BDI-II at 12 months |  |  |  |  |  |  |
| BAI at 6 months |  |  |  |  |  |  |
| BAI at 12 months |  |  |  |  |  |  |
| Smoking status at 6 months |  |  |  |  |  |  |
| Smoking status at 12 months |  |  |  |  |  |  |
| SGRQ at 6 months |  |  |  |  |  |  |
| SGRQ at 12 months |  |  |  |  |  |  |
| B-IPQ at 6 months |  |  |  |  |  |  |
| B-IPQ at 12 months |  |  |  |  |  |  |
| heiQ at 6 months |  |  |  |  |  |  |
| heiQ at 12 months |  |  |  |  |  |  |
| Time Use Survey at 6 months |  |  |  |  |  |  |
| Time Use Survey at 12 months |  |  |  |  |  |  |
| ZBI at 6 months |  |  |  |  |  |  |
| ZBI at 12 months |  |  |  |  |  |  |
| WEMWBS at 6 months |  |  |  |  |  |  |
| WEMWBS at 12 months |  |  |  |  |  |  |

Table 8 – Smoking status secondary outcome

|  | **Baseline**  No. (%) | | **6 months**  No. (%) | | **12 months**  No. (%) | |
| --- | --- | --- | --- | --- | --- | --- |
|  | **Intervention** | **Usual Care** | **Intervention** | **Usual Care** | **Intervention** | **Usual Care** |
| Current smoker |  |  |  |  |  |  |
| Non-smoker |  |  |  |  |  |  |
| Missing |  |  |  |  |  |  |

Table 9 – Results from sensitivity analyses for HADS-A at 6 months

|  | **Treatment effect (95% CI)** | **P-value** |
| --- | --- | --- |
| Main analysis |  |  |
| Complete case analysis |  |  |
| MNAR analysis – see Table 11 |  |  |
| Excluding participants with a score <8 on HADS-A subscale |  |  |
| Time to PR as a covariate |  |  |
| Excluding internal pilot participants |  |  |
| Meta-analysis combining results from the different allocation ratio periods |  |  |

Table 10 – Results from sensitivity analyses for HADS-D at 6 months

|  | **Treatment effect (95% CI)** | **P-value** |
| --- | --- | --- |
| Main analysis |  |  |
| Complete case analysis |  |  |
| MNAR analysis – see Table 12 |  |  |
| Excluding participants with a score <8 on HADS-D subscale |  |  |
| Time to PR as a covariate |  |  |
| Excluding internal pilot participants |  |  |
| Meta-analysis combining results from the different allocation ratio periods |  |  |

Table 11 - Results of sensitivity analysis for data being MNAR of HADS-A at 6 months

| **Assumed mean responses for participants with missing data in usual care group** | **Assumed mean responses for participants with missing data in intervention group** | **Treatment effect**  **(95% CI)** |
| --- | --- | --- |
| -10 | -15 |  |
|  | -10 |  |
|  | -5 |  |
| -5 | -10 |  |
|  | -5 |  |
|  | 0 |  |
| -1.5 | -6.5 |  |
|  | -1.5 |  |
|  | 3.5 |  |
| 0 | -5 |  |
|  | 0 |  |
|  | 5 |  |
| 1.5 | -3.5 |  |
|  | 1.5 |  |
|  | 6.5 |  |
| 5 | 0 |  |
|  | 5 |  |
|  | 10 |  |
| 10 | 5 |  |
|  | 10 |  |
|  | 15 |  |

Table 12 - Results of sensitivity analysis for data being MNAR of HADS-D at 6 months

| **Assumed mean responses for participants with missing data in usual care group** | **Assumed mean responses for participants with missing data in intervention group** | **Treatment effect**  **(95% CI)** |
| --- | --- | --- |
| -10 | -15 |  |
|  | -10 |  |
|  | -5 |  |
| -5 | -10 |  |
|  | -5 |  |
|  | 0 |  |
| -1.5 | -6.5 |  |
|  | -1.5 |  |
|  | 3.5 |  |
| 0 | -5 |  |
|  | 0 |  |
|  | 5 |  |
| 1.5 | -3.5 |  |
|  | 1.5 |  |
|  | 6.5 |  |
| 5 | 0 |  |
|  | 5 |  |
|  | 10 |  |
| 10 | 5 |  |
|  | 10 |  |
|  | 15 |  |

Table 13 – CBA and PR attendance and completion

|  | **CBA intervention** | **PR** |
| --- | --- | --- |
| Attendance rate |  |  |
| Completion rate |  |  |

**Abbreviations: CBA**: Cognitive Behavioural Approach; **PR**: Pulmonary Rehabilitation

Table 14 – Adverse events

|  | **Intervention**  **N (%)** | **Usual care**  **N (%)** |
| --- | --- | --- |
| **Reporting method**  Event known to researcher during patient assessment  Event know to facilitator during intervention delivery |  |  |
| **Severity**  Mild  Moderate  Severe  Life threatening  Death |  |  |
| **Causality**  Unrelated  Unlikely  Possibly  Probably  Definitely |  |  |
| **Action taken**  No action taken  Withdrawal  Con Med  Non-drug therapy  Hospitalisation |  |  |
| **Outcome**  Unresolved  Resolving  Resolved  Fatal  Unknown |  |  |
| **Expectedness**  Expected  Unexpected |  |  |

Table 15 – Serious adverse events

|  | **Intervention**  **N (%)** | **Usual care**  **N (%)** |
| --- | --- | --- |
| **Reporting method**  Event known to researcher during patient assessment  Event know to facilitator during intervention delivery |  |  |
| **Severity**  Results in death  Life threatening  Hospitalisation or prolongation of hospitalisation  Persistent or significant disability or incapacity  Congenital anomaly or birth defect  “Other” important medical event |  |  |
| **Related to one of the procedures in the study**  Related  Unrelated |  |  |
| **Expectedness**  Expected  Unexpected |  |  |
| **Due to the progression of an underlying illness**  Yes  No |  |  |
| **Action taken with study treatment and procedures**  Continued  Reduced  Increased  Temporary stop  Permanent stop |  |  |
| **Related to the trial conduct**  Yes  No  Not answered |  |  |
| **PI withdrew the patient from the study**  Yes  No |  |  |
| **Outcome**  Resolved  Resolved with sequelae  Improved  Persisting  Worsened  Fatal  Unknown |  |  |
